# Diagnostic Value of NO-Related Biomarkers (ADMA, NO, eNOS) in Stable COPD and Acute Exacerbation of COPD

**DOI:** 10.3390/jcm14207386

**Published:** 2025-10-19

**Authors:** Osman El Jundi, Aysen Kutan Fenercioglu, Pelin Uysal, Seyma Dumur, Oguzhan Cucu, Hafize Uzun

**Affiliations:** 1 Department of Chest Diseases, Faculty of Medicine, Istanbul Atlas University, Istanbul 34403, Turkey; osmaneljundi2020@gmail.com (O.E.J.); drpelinuysal@gmail.com (P.U.); 2 Department of Family Medicine, Cerrahpasa Medical Faculty, Istanbul University-Cerrahpasa, Istanbul 34098, Turkey; aysenfenerci@hotmail.com; 3 Department of Chest Diseases, Medicana Zincirlikuyu Hospital, Istanbul 34394, Turkey; 4 Department of Medical Biochemistry, Faculty of Medicine, Istanbul Atlas University, Istanbul 34403, Turkey; seyma_dumur@hotmail.com; 5 Department of Anaesthesiology and Intensive Care, Medicana Zincirlikuyu Hospital, Istanbul 34394, Turkey; oguzhancucu@medicana.com.tr

**Keywords:** acute exacerbation, asymmetric dimethylarginine, endothelial nitric oxide synthase, nitric oxide, stable COPD

## Abstract

**Background:** Nitric oxide (NO)-related biomarkers, including asymmetric dimethylarginine (ADMA), nitric oxide (NO), and endothelial nitric oxide synthase (eNOS), may play a role in the pathophysiology and clinical progression of chronic obstructive pulmonary disease (COPD). This study aimed to investigate their diagnostic value in stable COPD and acute exacerbation. **Methods:** A total of 150 participants (76 females, 74 males; mean age 53.82 ± 7.06 years) were enrolled and equally distributed into control, stable COPD, and acute exacerbation groups (*n* = 50 each). Demographic, clinical, and laboratory parameters were compared across groups. Serum ADMA, NO, and eNOS levels were analyzed, and correlations with clinical findings were evaluated. ROC analysis was performed to determine the diagnostic performance of the biomarkers. **Results:** Serum ADMA levels were significantly higher in COPD patients, particularly in the acute exacerbation group (*p* < 0.05 for all comparisons). In contrast, NO and eNOS levels were significantly lower in COPD groups compared to controls (*p* < 0.05 for all comparisons). ADMA showed strong negative correlations with FEV_1_, FEV_1_/FVC, PaO_2_, and SaO_2_, whereas NO and eNOS showed positive correlations with the same parameters (all *p* < 0.01). For predicting acute exacerbation, an ADMA cut-off of 1.36 yielded high diagnostic accuracy (AUC = 0.983; sensitivity 86.0%; specificity 96.0%). eNOS also demonstrated predictive value (AUC = 0.823). For stable COPD, NO at a cut-off of 14.91 showed excellent diagnostic performance (AUC = 0.921). **Conclusions:** NO-related biomarkers, particularly ADMA and NO, may serve as reliable indicators for differentiating between stable COPD and acute exacerbation. Elevated ADMA and reduced NO and eNOS levels were closely associated with impaired lung function and oxygenation parameters. These findings suggest potential clinical utility of these biomarkers in COPD monitoring and management.

## 1. Introduction

Chronic obstructive pulmonary disease (COPD) represents one of the leading causes of illness and death globally. It is defined by a persistent limitation of airflow and ongoing airway inflammation, most commonly resulting from prolonged exposure to cigarette smoke or other harmful particles and gases [1,2,3]. The worldwide prevalence of COPD has been estimated at around 10.6% [4]. According to the World Health Organization (WHO), COPD ranked as the fourth leading cause of death in 2021, accounting for approximately 3.5 million deaths across the globe [5]. In its advanced stages, the disease is often complicated by acute exacerbations (AECOPDs), periods of symptom intensification that markedly worsen patients’ health outcomes and increase the risk of hospitalization and mortality [6].

A well-established complication of COPD is the development of pulmonary hypertension (PH), which significantly contributes to disease progression and worsened clinical outcomes [7]. The increase in pulmonary arterial pressure is largely attributed to the reduced activity of nitric oxide (NO), a potent endogenous vasodilator involved in regulating vascular tone. NO produced by the endothelial isoform of nitric oxide synthase (eNOS) in endothelial cells (ECs) is the central regulator of vascular tone and systemic hemodynamics [8]. Under chronic hypoxic conditions, secondary PH is frequently observed, accompanied by increased pulmonary expression of eNOS enzymes, reflecting a maladaptive response to impaired NO bioavailability [9]. The activity of eNOS, the enzyme responsible for NO synthesis, is regulated by an endogenous inhibitor, asymmetric dimethylarginine (ADMA). It is well established that ADMA modulates the rate of nitric oxide production [9].

Following the full development of emphysema, inhibition of inducible nitric oxide synthase (iNOS) has been associated with the partial restoration of lung architecture and an improvement in pulmonary function [10]. Nevertheless, the specific involvement of the asymmetric dimethylarginine (ADMA)/nitric oxide (NO) axis in this process remains poorly understood. Elevated ADMA levels have been detected in the exhaled breath condensate of individuals with COPD [11], implying that ADMA may play a regulatory role in NO bioavailability, an association that warrants further investigation. Moreover, evidence suggests that endothelial nitric oxide synthase (eNOS) in the circulation may contribute to the control of plasma nitrite concentrations and blood pressure homeostasis [12]; however, its precise role has not yet been clearly defined. Based on previous evidence of dysregulation in the NO pathway in COPD (10, 11), we hypothesized that serum levels of NO-related biomarkers (ADMA, NO, and eNOS) differ between healthy individuals, patients with stable COPD, and those with acute exacerbations, and may serve as diagnostic indicators of disease stage. Therefore, this study aimed to investigate the diagnostic value of serum ADMA, NO, and eNOS levels in COPD, with particular emphasis on their role in differentiating stable disease from acute exacerbations. Additionally, correlations with lung function and oxygenation parameters were assessed to gain insight into their potential clinical utility.

## 2. Materials and Methods

### 2.1. Ethical Approval

This study was conducted using a prospective design. All procedures were performed in accordance with the ethical standards of the 1964 Declaration of Helsinki and its later amendments or comparable ethical principles. Ethical approval was obtained from the Mehmet Ali Aydınlar University Faculty of Medicine Ethics Committee (approval number: ATADEK-2020/02; approval date: 11 February 2020), and written informed consent was obtained from all participants before enrollment.

### 2.2. Study Design and Population

This cross-sectional study included 150 participants, who were evenly distributed into three groups: healthy controls (*n* = 50), patients with stable COPD (*n* = 50), and patients experiencing an acute exacerbation of COPD (*n* = 50). The diagnosis of COPD was established based on the criteria outlined by the Global Initiative for Chronic Obstructive Lung Disease (GOLD) [13].

### 2.3. Definition of AECOPD

Acute exacerbation was defined as a sustained worsening of respiratory symptoms requiring treatment modification, based on the Anthonisen criteria, which comprise three patient-reported items, namely, increased dyspnea, increased sputum volume, and increased sputum purulence [14,15].

### 2.4. Definition of Stable COPD

The stable phase of COPD was defined as the period occurring 3–4 weeks after an exacerbation, during which acute symptoms had resolved and patients had returned to their baseline levels of dyspnea, cough, and sputum production, showing only normal daily fluctuations. To qualify as stable, patients must not have experienced any exacerbation or required antibiotics for infection within the previous six weeks, and there should have been no change in exercise tolerance, sputum characteristics, or treatment regimen, including oral corticosteroid use, in the preceding 2 weeks.

### 2.5. Inclusion Criteria

A post-bronchodilator FEV1 of less than 80% of the predicted value, together with an FEV1/FVC ratio below 70%, was accepted as an indicator of airflow limitation [13]. In COPD patients, increased dyspnea, sputum volume, and sputum purulence were considered criteria for exacerbation [15]. For patients classified as having stable COPD, the absence of an exacerbation within the last six weeks and no use of antibiotics for any infection were required.

### 2.6. Exclusion Criteria

Patients with another concomitant respiratory disease (such as pulmonary embolism, pulmonary tuberculosis, bronchial asthma, bronchiectasis, interstitial lung disease, pneumonia, or lung cancer), as well as those with heart failure, diabetes mellitus, malignancy, sepsis, chronic renal failure, coronary artery disease, rheumatologic disorders, lower extremity problems preventing ambulation, or inability to comply with pulmonary function testing, were excluded from the study.

### 2.7. Clinical and Demographic Assessment

Demographic data (age, sex, BMI), smoking exposure (pack-years), and duration of disease were recorded. Blood pressure measurements were obtained under resting conditions. Pulmonary function tests, including FEV_1_ (% predicted) and FEV_1_/FVC ratio, were performed using a standardized spirometer. Arterial blood gas analysis was conducted to determine partial oxygen pressure (PaO_2_) and oxygen saturation (SaO_2_).

### 2.8. Pulmonary Function Tests

Pulmonary function tests were performed using desktop spirometer (Cosmed/Pony Fx, Rome, Italy) with patients in a 90° upright sitting position after having been previously instructed on the procedure. The tests were conducted according to the American Thoracic Society (ATS) and the European Respiratory Society (ERS) criteria, with at least three technically acceptable maneuvers required [16]. From the three obtained flow-volume curves, the highest values of FVC and FEV_1_ were selected.

### 2.9. Assessment of Blood Tests

Routine blood tests (including leukocyte count and differential leukocyte count, hemoglobin, and platelet count) were carried out using a Sysmex XN-1000 automated hematology analyzer (Sysmex XN-1000 Corporation, HQ: Kobe, Japan).

Levels of rutin biochemical parameters were assessed using the spectrophotometric method by the autoanalyzer (Hitachi Modular System, Roche Diagnostic, Boston, MA, USA). C-reactive protein (CRP) values were measured with the turbidimetric method by analyzer (ADVIA 1800 Auto Analyzer, Siemens Medical Sol., Deerfield, IL, USA).

Serum concentrations of ADMA, NO, and eNOS were measured using commercially available enzyme-linked immunosorbent assay (ELISA) kits: Human Asymmetrical Dimethylarginine (ADMA) ELISA Kit, Cat. No. MBS161697 (MyBioSource, San Diego, CA, USA), Human Nitric Oxide (NO) ELISA Kit, Cat. No. MBS2606568 (MyBioSource, San Diego, CA, USA), Human Endothelial Nitric Oxide Synthase (eNOS) ELISA Kit, Cat. No. MBS265088 (MyBioSource, San Diego, CA, USA). All analyses were performed according to the manufacturers’ protocols. The intra-assay and inter-assay coefficients of variation (CVs) for these kits were <10% and <12%, respectively.

### 2.10. Statistical Analysis

Statistical analyses of the data obtained in this study were performed using *NCSS (Number Cruncher Statistical System) 2020 Statistical Software* (NCSS LLC, Kaysville, UT, USA). Descriptive statistical methods were used to present the data; quantitative variables were expressed as mean, standard deviation, median, minimum, and maximum values, while categorical variables were presented as frequency and percentage. The normality of the data distribution was assessed using the Shapiro–Wilk test and Box Plot graphs. For comparisons between two groups of normally distributed quantitative variables, Student’s *t*-test was used. For comparisons among three or more groups with normally distributed quantitative variables, the One-Way ANOVA test was applied, and the Bonferroni test was used to determine the source of differences. For comparisons of ADMA, eNOS, and NO levels between groups after adjusting for the effect of BMI, the ANCOVA test was performed, followed by the Bonferroni post hoc test to identify pairwise differences. For non-normally distributed variables, comparisons among three or more groups were made using the Kruskal–Wallis test, and pairwise comparisons were performed with the Dunn test. Correlations between variables were evaluated using Pearson’s correlation analysis according to the data distribution. For ADMA, NO, and eNOS measurements, partial (adjusted) correlation analyses were performed after adjusting for the effect of BMI.

The Pearson’s Chi-square test was used to compare qualitative data. Receiver Operating Characteristic (ROC) curve analysis was performed to evaluate the diagnostic performance of ADMA and eNOS for predicting the presence of acute exacerbation, and of ADMA, NO, and eNOS for predicting stable COPD. Differences between AUC values were assessed using the DeLong test.

All results were evaluated within a 95% confidence interval, and a *p*-value < 0.05 was considered statistically significant.

## 3. Results

### 3.1. Characteristics of the Study Populations

The study was conducted with a total of 150 subjects, comprising 50.7% (*n* = 76) females and 49.3% (*n* = 74) males. The ages of the participants ranged from 35 to 68 years, with a mean of 53.82 ± 7.06 years. Body mass index (BMI) values ranged from 19.6 to 40.6, with a mean of 27.21 ± 4.28. The duration of COPD varied between 5.4 and 11.3 years, with a mean of 8.10 ± 1.50 years. Smoking exposure (pack-years) ranged from 18.6 to 110.5, with a mean of 54.42 ± 21.95.

Regarding the frequency of COPD exacerbations, 58% (*n* = 29) of the participants reported no exacerbations, 22% (*n* = 11) experienced one exacerbation, and 20% (*n* = 10) had two or more exacerbations. The distribution across groups was equal, with each group (control, stable, and acute exacerbation) comprising 33.3% (*n* = 50) of the total sample. FEV_1_ (% predicted) values ranged from 20.5 to 89.3, with a mean of 61.31 ± 20.2. The FEV_1_/FVC ratio ranged from 38 to 90, with a mean of 68.61 ± 12.95. Partial pressure of oxygen (PaO_2_) values ranged from 42.9 to 100 mmHg, with a mean of 68.71 ± 15.42 mmHg. Oxygen saturation (SaO_2_) values ranged from 65.4% to 97.6%, with a mean of 84.17 ± 9.16%.

### 3.2. The Comparison of Demographic and Clinical Findings

Table 1 shows the comparison of demographic and clinical findings. There were no differences between the groups in terms of age and gender (*p* > 0.05). The BMI values in the control group were significantly lower than those in the stable COPD and acute exacerbation groups (*p* = 0.001; *p* = 0.001; *p* < 0.01). Systolic blood pressure values did not differ significantly between the groups (*p* > 0.05). However, diastolic blood pressure was significantly higher in the acute exacerbation group compared to both the control and stable COPD groups (*p* = 0.001; *p* = 0.001; *p* < 0.01). FEV_1_ (% predicted) values also showed significant differences among the groups (*p* = 0.001; *p* < 0.01). FEV_1_ values in the control group were significantly higher than those in the stable COPD and acute exacerbation groups (*p* = 0.001; *p* = 0.001; *p* < 0.01), and values in the stable COPD group were significantly higher than those in the acute exacerbation group (*p* = 0.031; *p* < 0.05) (Table 1).

FEV_1_/FVC ratios showed differences among the groups (*p* = 0.001; *p* < 0.01). The FEV_1_/FVC ratios in the control group were significantly higher than those in both the stable COPD and Acute Exacerbation groups (*p* = 0.001; *p* = 0.001; *p* < 0.01). Additionally, the stable COPD group had significantly higher FEV_1_/FVC ratios than the acute exacerbation group (*p* = 0.001; *p* < 0.01). PaO_2_ (mmHg) values differed significantly between the groups (*p* = 0.001; *p* < 0.01). PaO_2_ levels in the control group were significantly higher than those in both the stable COPD and acute exacerbation groups (*p* = 0.001; *p* = 0.001; *p* < 0.01). Furthermore, PaO_2_ levels in the stable COPD group were significantly higher than those in the acute exacerbation group (*p* = 0.038; *p* < 0.05). SaO_2_ (%) values also showed differences among the groups (*p* = 0.001; *p* < 0.01). Oxygen saturation values in the control group were significantly higher than those in both the Stable COPD and acute exacerbation groups (*p* = 0.001; *p* = 0.001; *p* < 0.01) (Table 1). The pH and pCO_2_ values also differed significantly among the groups (*p* = 0.001; *p* < 0.01). The pH values in the control group were higher than those in the stable COPD and acute exacerbation groups (*p* = 0.001; *p* = 0.001; *p* < 0.01), whereas the pH values in the stable COPD group were higher than those in the acute exacerbation group (*p* = 0.001; *p* < 0.01). The PCO_2_ values in the control group were lower than those in the stable COPD and acute exacerbation groups (*p* = 0.001; *p* = 0.001; *p* < 0.01), while the PCO_2_ values in the stable COPD group were lower than those in the acute exacerbation group (*p* = 0.001; *p* < 0.01) (Table 1).

### 3.3. The Comparison of Laboratory Findings, Serum ADMA, NO, and eNOS Levels

Table 2 shows the comparison of laboratory findings together with serum ADMA, NO, and eNOS levels. Insulin and HOMA-IR values did not show significant differences between the groups. However, serum levels of CRP, ESR, albumin, triglycerides, LDL, and total cholesterol demonstrated differences among the groups (Table 2).

After controlling for BMI as a covariate, ADMA values differed significantly among the groups (F = 48.636; *p* = 0.001; *p* < 0.01). The ADMA levels in the acute exacerbation group were significantly higher than those in the control and stable COPD groups (*p* = 0.001; *p* = 0.001; *p* < 0.01) (Table 2).

After controlling for BMI as a covariate, NO values showed a statistically significant difference among the groups (F = 113.135; *p* = 0.001; *p* < 0.01). The NO levels in the acute exacerbation group were significantly lower than those in the control and stable COPD groups (*p* = 0.001; *p* = 0.001; *p* < 0.01), and the NO levels in the stable COPD group were also lower than those in the Control group (*p* = 0.001; *p* < 0.01) (Table 2).

After controlling for BMI as a covariate, eNOS values differed significantly among the groups (F = 15.385; *p* = 0.001; *p* < 0.01). The eNOS levels in both the acute exacerbation and stable COPD groups were significantly lower than those in the control group (*p* = 0.001; *p* = 0.001; *p* < 0.01) (Table 2).

### 3.4. Correlation Analysis Between the Parameters

Table 3 shows the correlation analysis between NO-related biomarkers and laboratory and clinical findings in COPD patients. After adjusting for BMI as a covariate, no statistically significant correlation was found between age and ADMA levels (*p* > 0.05). A positive correlation was observed between BMI and ADMA levels (r = 0.265; *p* = 0.001; *p* < 0.01). No correlation was found between COPD duration and ADMA levels (*p* > 0.05). Negative correlations were found between FEV_1_ and ADMA levels (r = –0.481; *p* = 0.001; *p* < 0.01) and the FEV_1_/FVC ratio and ADMA levels (r = –0.293; *p* = 0.003; *p* < 0.01). Furthermore, PaO_2_ was negatively correlated with ADMA (r = –0.209; *p* = 0.038; *p* < 0.05), and a significant negative correlation was also found between SaO_2_ and ADMA levels (r = –0.272; *p* = 0.006; *p* < 0.01). While a significant negative correlation was found between pH and ADMA levels (r = –0.419; *p* = 0.001; *p* < 0.01), a positive correlation was observed between PCO_2_ and ADMA levels (r = 0.393; *p* = 0.001; *p* < 0.01) (Table 3).

After adjusting for BMI as a covariate, no statistically significant correlation was found between age and NO levels (*p* > 0.05). In contrast, a negative correlation was observed between BMI and NO levels (r = –0.430; *p* = 0.001; *p* < 0.01). No significant association was found between the duration of COPD and NO levels (*p* > 0.05). While a positive correlation was found between FEV_1_ and NO levels (r = 0.286; *p* = 0.004; *p* < 0.01), no significant correlation was observed between the FEV_1_/FVC ratio and NO levels (*p* > 0.05). Similarly, there was no significant correlation between PaO_2_ and NO levels (*p* > 0.05), but a positive correlation was detected between SaO_2_ and NO levels (r = 0.345; *p* = 0.001; *p* < 0.01). In addition, a significant positive correlation was found between pH and NO levels (r = 0.366; *p* = 0.001; *p* < 0.01), whereas a significant negative correlation was observed between PCO_2_ and NO levels (r = –0.418; *p* = 0.001; *p* < 0.01) (Table 3).

After adjusting for BMI as a covariate, no statistically significant correlations were found between eNOS levels and age, BMI, and duration of COPD (*p* > 0.05). A positive correlation was observed between FEV_1_ and eNOS levels (r = 0.372; *p* = 0.001; *p* < 0.01), but no correlation was found between the FEV_1_/FVC ratio and eNOS levels (*p* > 0.05). Similarly, PaO_2_ and SaO_2_ showed no correlations with eNOS levels (*p* > 0.05). No significant correlation was detected between pH and eNOS levels (*p* > 0.05), whereas a negative correlation was found between PCO_2_ and eNOS levels (r = –0.211; *p* = 0.036; *p* < 0.05) (Table 3).

### 3.5. The Comparisons by Gender and Exacerbation Frequency

After controlling for BMI as a covariate, no statistically significant differences were found in ADMA levels between males and females (*p* > 0.05). Similarly, NO and eNOS levels did not differ significantly between sexes after adjusting for BMI (*p* > 0.05) (Table 4).

After controlling for BMI as a covariate, no statistically significant differences were observed in ADMA levels among the frequency-of-exacerbation groups (*p* > 0.05). Likewise, NO and eNOS levels showed no significant differences among these groups after adjusting for BMI (*p* > 0.05) (Table 4).

### 3.6. Diagnostic Value of NO-Related Biomarkers for Predicting Acute Exacerbation of COPD

**Figure 1:**
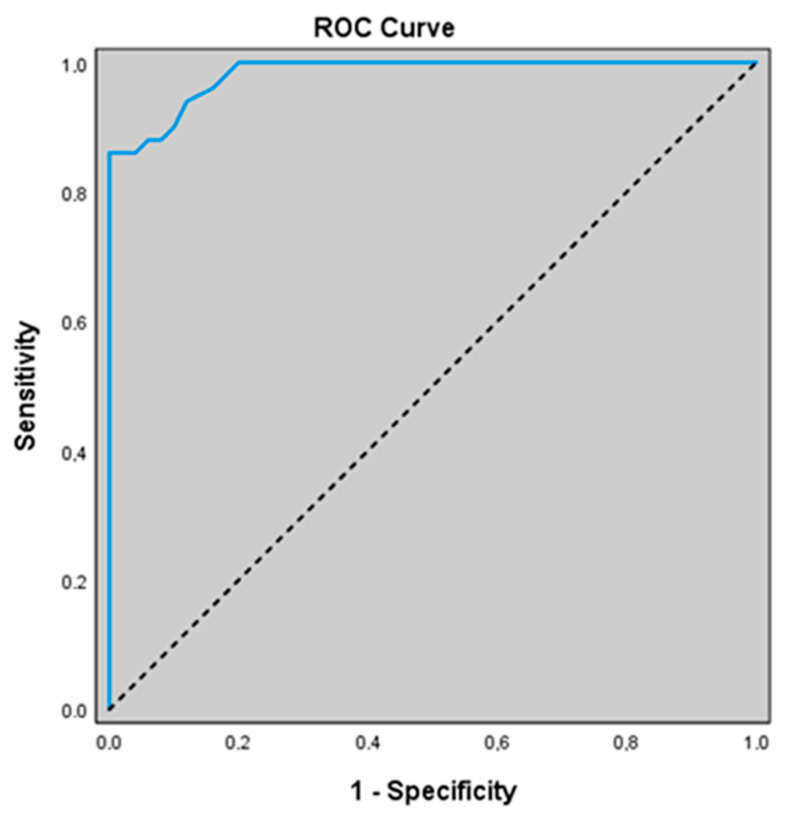
ROC curve of ADMA levels for predicting the presence of acute exacerbation.

In our study, for predicting the presence of acute exacerbation, the ADMA level at a cut-off value of 1.36 showed a sensitivity of 86.0%, specificity of 96.0%, positive predictive value (PPV) of 95.6%, and negative predictive value (NPV) of 87.3%. The area under the ROC curve (AUC) was calculated as 0.983 with a standard error of 0.09. An association was found between ADMA levels ≥ 1.36 and the presence of acute exacerbation (*p* = 0.000; *p* < 0.01). The odds of having an acute exacerbation were 147.429 times higher in patients with ADMA levels ≥1.36. The odds ratio (OR) for ADMA was 147.429 (95% CI: 29.046–748.301) (Table 5) (Figure 1).

**Figure 2:**
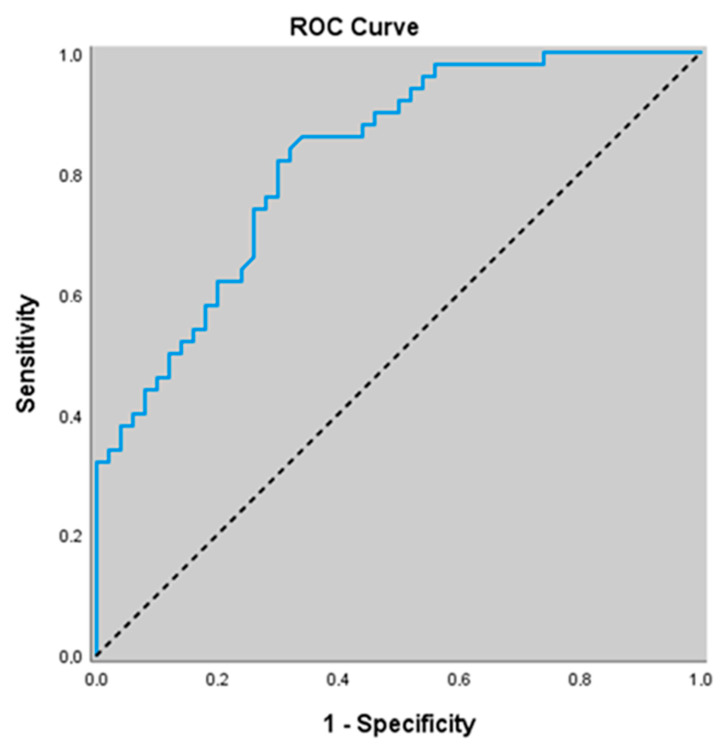
ROC curve of eNOS levels for predicting the presence of acute exacerbation.

Similarly, for predicting the presence of acute exacerbation, an eNOS cut-off value of 44.52 yielded a sensitivity of 82.0%, specificity of 70.0%, positive predictive value (PPV) of 73.2%, and negative predictive value (NPV) of 79.5%. The area under the ROC curve (AUC) was calculated as 0.823 with a standard error of 0.040. An association was found between eNOS levels ≤ 44.52 and the presence of acute exacerbation (*p* = 0.000; *p* < 0.01). The risk of detecting acute exacerbation was 10.630 times higher in patients with eNOS levels ≤ 44.52. The odds ratio (OR) for eNOS was 10.630 (95% CI: 4.146–27.252) (Table 5) (Figure 2).

The difference between the AUC values of adma (µmol/L) and enos (µmol/L) was statistically significant (*p* < 0.01), indicating that adma demonstrated superior diagnostic performance in distinguishing acute exacerbation.

### 3.7. Diagnostic Value of Non-Related Biomarkers for Predicting Stable COPD

**Figure 3:**
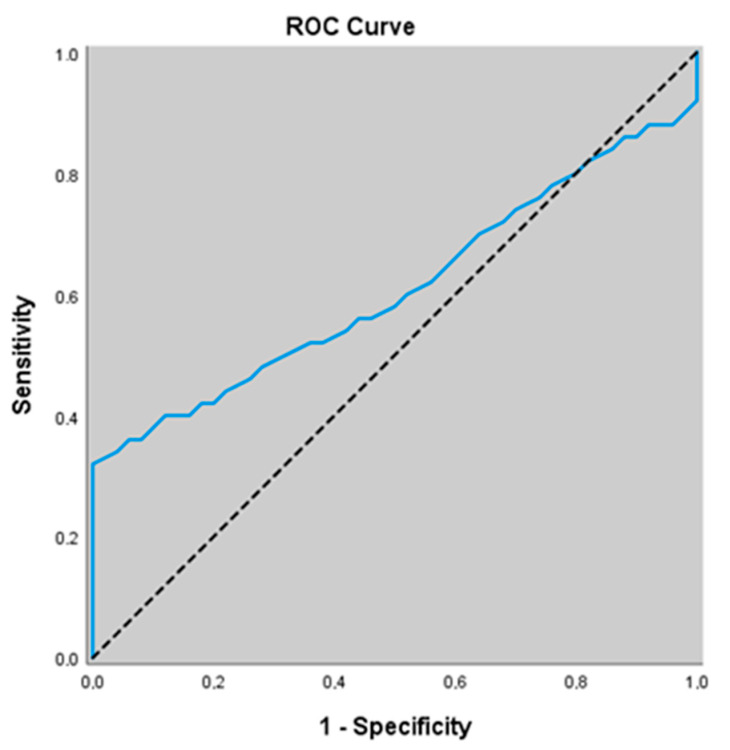
ROC curve of ADMA levels for predicting the presence of stable COPD.

In our study, for predicting the presence of stable COPD, the ADMA level at a cut-off value of 1.36 showed a sensitivity of 34.0%, specificity of 96.0%, positive predictive value (ppv) of 89.5%, and negative predictive value (npv) of 59.3%. the area under the ROC curve (AUC) was calculated as 0.609 with a standard error of 0.058. There was no association between ADMA levels ≥ 1.36 and the presence of stable COPD (*p* = 0.062; *p* > 0.05) (Table 6, Figure 3).

**Figure 4:**
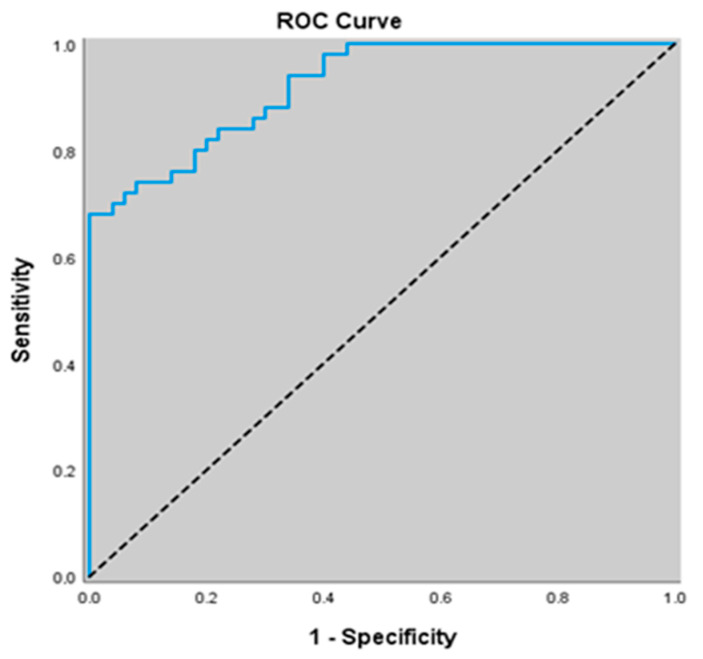
ROC curve of NO levels for predicting the presence of stable COPD.

Similarly, for predicting the presence of stable COPD, an NO cut-off value of 14.91 yielded a sensitivity of 68.0%, specificity of 100.0%, positive predictive value (PPV) of 100.0%, and negative predictive value (NPV) of 75.8%. The area under the ROC curve (AUC) was calculated as 0.921 with a standard error of 0.025. An association was found between NO levels ≤14.91 and the presence of stable COPD (*p* = 0.000; *p* < 0.01). The risk of detecting stable COPD was 0.242 times higher in patients with NO levels ≤ 14.91. The odds ratio (OR) for NO was 0.242 (95% CI: 0.158–0.371) (Table 5) (Figure 4).

**Figure 5:**
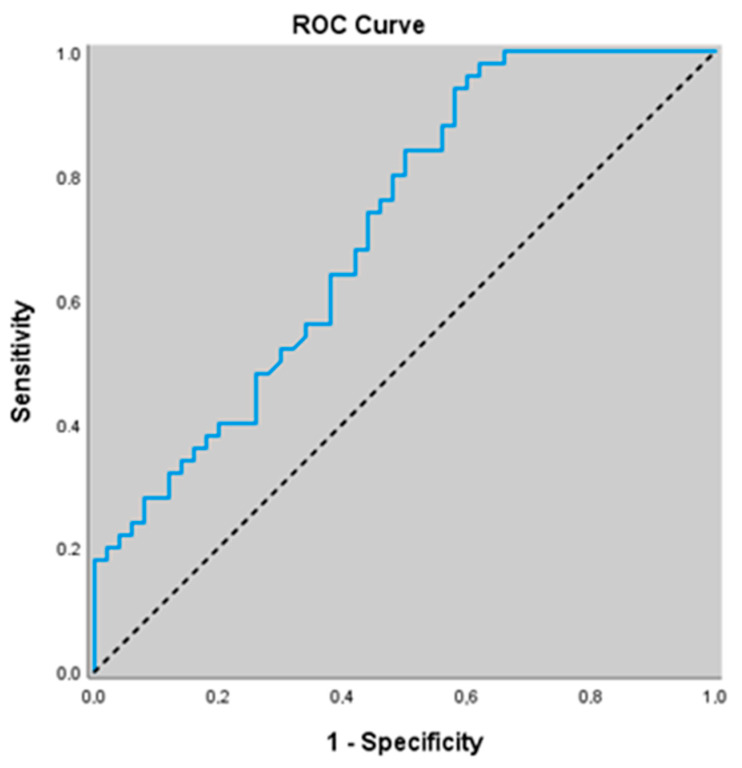
ROC curve of eNOS levels for predicting the presence of stable COPD.

Furthermore, for predicting the presence of stable COPD, an eNOS cut-off value of 48.8 yielded a sensitivity of 94.0%, specificity of 42.0%, positive predictive value (PPV) of 61.8%, and negative predictive value (NPV) of 87.5%. The area under the ROC curve (AUC) was calculated as 0.713 with a standard error of 0.051. An association was found between eNOS levels ≤ 48.8 and the presence of stable COPD (*p* = 0.000; *p* < 0.01). The risk of detecting stable COPD was 11.345 times higher in patients with eNOS levels ≤ 48.8. The odds ratio (OR) for eNOS was 11.345 (95% CI: 3.107–41.429) (Table 5 and Figure 5).

Significant differences were observed among the AUC values of ADMA, NO, and eNOS (all *p* < 0.01). NO exhibited superior diagnostic performance in distinguishing stable COPD compared with ADMA and eNOS (both *p* < 0.01).

## 4. Discussion

The most important finding of the present study is that serum ADMA, NO, and eNOS levels were significantly altered in patients with COPD compared with healthy controls, showing distinct differences between stable and exacerbation phases. Specifically, ADMA levels were progressively higher in patients with stable COPD and reached their peak during acute exacerbations, whereas NO and eNOS levels were highest in healthy controls and gradually decreased in patients with stable and exacerbated COPD. This pattern suggests a dysregulation of the ADMA/NO axis in COPD, which may contribute to endothelial dysfunction, impaired vasodilation, and worsening pulmonary function.

Elevated serum iNOS and eNOS levels in AECOPD patients were correlated with 30-day readmission, and the combined assessment of Hb, Alb, CRP, NOS levels, and APACHE II score within 24 h of admission effectively predicted readmission risk [17]. Duan and Cheng [18] reported that in AECOPD patients complicated by depression, serum neuroactive substances, including NO, were significantly altered, with NO levels increased. NO levels were correlated with disease severity and the frequency of exacerbations, suggesting their potential diagnostic value. Csoma et al. [19] demonstrated that dysregulation of the endothelial nitric oxide (NO) pathway is linked to airway inflammation in COPD. Their study highlights that impaired NO bioavailability can contribute to endothelial dysfunction and may play a significant role in the pathophysiology of chronic airway inflammation in these patients. Consistent with our findings, this study supports the role of NO dysregulation in COPD pathophysiology and highlights the broader relevance of neuroactive substances, including the ADMA/NO axis, in disease complications.

Hannemann et al. [20] reported that the L-arginine/dimethylarginine/nitric oxide (NO) pathway is disrupted in chronic respiratory diseases, particularly in association with intermittent hypoxemia. In patients with COPD accompanied by intermittent hypoxemia, plasma levels of asymmetric (ADMA) and symmetric dimethylarginine (SDMA) were found to be elevated. This alteration may contribute to reduced pulmonary NO synthesis, increased pulmonary vasoconstriction, and subsequent disease progression. The authors emphasized the need for future prospective studies to confirm these findings and clarify the underlying mechanisms. These findings are consistent with our research, in which ADMA and NO levels reflected differences between stable COPD and acute exacerbations, and correlated with impaired pulmonary function. Hannemann et al.’s [20] results further support the notion that elevated ADMA and SDMA can disrupt NO bioavailability, adversely affecting disease courses in patients with combined airway obstruction and sleep-disordered breathing. Overall, these data highlight the potential role of ADMA and related biomarkers in understanding the pathophysiology and prognosis of COPD and suggest their possible utility in clinical practice for risk stratification and disease monitoring. Elevated ADMA concentrations may serve as an additional indicator for identifying the presence of pulmonary hypertension (PH). Therapeutic strategies aimed at reducing ADMA levels could offer potential benefits for patients with COPD-associated PH. Consequently, ADMA-lowering interventions may represent a promising novel therapeutic target for the prevention and management of PH [20]. These findings align with our study, where dysregulation of the ADMA/NO axis was associated with impaired vascular function and disease severity in COPD patients. Telo et al.’s [21] work suggests that elevated ADMA may inhibit NO bioavailability, impair endothelial function, and contribute to the development of PH in COPD. Therefore, serum ADMA levels could serve as a useful tool for the early detection and prognostic assessment of PH in patients with COPD. However, further prospective studies are needed to validate this mechanism and its clinical utility.

In patients with COPD, serum ADMA levels are elevated, whereas NO concentrations are reduced. Both ADMA and NO show strong and independent associations with the degree of airflow limitation, as indicated by FEV_1_ measurements. These observations suggest that ADMA, as an endogenous NOS inhibitor, may contribute to systemic inflammation and airway remodeling in COPD. Therefore, therapeutic strategies aimed at lowering ADMA levels could represent a promising approach for COPD management. Further longitudinal studies are warranted to determine whether ADMA levels continue to rise with disease progression and to assess their potential value in predicting exacerbations, comorbidities, and mortality among COPD patients [22]. When analyzed according to disease severity, only patients with moderate COPD exhibited values above the normal range, whereas those with mild disease remained within normal limits, suggesting that alterations in ADMA and arginine metabolism become more pronounced as the disease progresses. These observations indicate that exacerbation phases may be associated with increased oxidative stress and a higher ADMA/arginine ratio, potentially driven by oxidative mechanisms. Further research involving larger cohorts that encompass all stages of COPD is needed to comprehensively elucidate the influence of arginine and ADMA on disease progression and deterioration [23].

Studies examining the relationship between NO and pulmonary function in COPD have reported conflicting results. Corradi et al. [24] observed increased exhaled NO in stable COPD patients, positively correlating with FEV_1_. In contrast, another study reported no difference in NO levels between COPD patients and healthy controls and found an inverse correlation with FEV_1_ [25]. Similarly, Rutgers et al. [26] reported that NO metabolism was not altered in stable COPD patients. This study on Tunisian COPD patients demonstrated significant alterations in oxidative status, with decreased plasma NO levels. Multivariate analysis indicated that NO is linked to pathological pathways affecting outcomes independently of airflow limitation, while peroxynitrite may contribute to bronchodilation, as suggested by its correlation with FEV_1_/FVC. These findings highlight an imbalance in the oxidant–antioxidant system in COPD and suggest that assessing oxidative status and considering antioxidant therapies may be valuable for management, though further research is needed to clarify the roles of NO and peroxynitrite in disease pathogenesis [27]. In another study, lower serum NO levels were reported in patients with COPD [28]. According to the literature, reduced NO levels in COPD may result from hypoxia, mucus hypersecretion, or decreased NO synthase expression, which has been linked to pulmonary vascular changes rather than airway obstruction severity [29]. Additionally, the high concentrations of NO present in cigarette smoke may interfere with endogenous NO production by suppressing eNOS activity [30]. In the present study, NO-related biomarkers demonstrated distinct predictive patterns for stable COPD and acute exacerbations. For acute exacerbations, an ADMA cut-off of 1.36 showed high sensitivity (86.0%) and specificity (96.0%), with a strong significant association and an odds ratio of 147.43, while an eNOS cut-off of 44.52 showed good sensitivity (82.0%) and moderate specificity (70.0%) with an odds ratio of 10.63, indicating that elevated ADMA and decreased eNOS are strongly linked to acute episodes. In contrast, for stable COPD, ADMA had limited predictive value, with low sensitivity (34.0%) despite high specificity (96.0%) and no significant association. NO at a cut-off of 14.91 showed excellent specificity (100.0%) and good sensitivity (68.0%), while eNOS at 48.8 showed high sensitivity (94.0%) but moderate specificity (42.0%), both significantly associated with stable COPD. These findings indicate that while ADMA is a strong biomarker for acute exacerbations, NO and eNOS may serve as more reliable indicators for identifying stable COPD, highlighting the differential role of the ADMA/NO/eNOS axis across disease phases.

### Limitations of the Study

This study has several limitations that should be acknowledged. First, the relatively small sample size may limit the generalizability of the findings to broader COPD populations. Second, the cross-sectional design prevents the establishment of causal relationships between ADMA, NO, eNOS levels, and COPD progression. Third, potential confounding factors such as diet, medication use, and environmental exposures were not fully controlled, which may have influenced biomarker levels. Furthermore, inhaled maintenance therapies (LABA, LAMA, ICS) and the use of roflumilast (a PDE-4 inhibitor that may influence NO metabolism) were not recorded in our dataset. This prevented us from evaluating potential interactions between pharmacological treatment and NO-related biomarkers. Finally, the absence of longitudinal follow-up data restricts the ability to assess the prognostic value of these biomarkers over time.

## 5. Conclusions

In conclusion, this study demonstrates that alterations in ADMA, NO, and eNOS levels are associated with different stages of COPD, with significant discriminative potential particularly for acute exacerbations. While ADMA showed a strong predictive value for exacerbation risk, NO and eNOS were more closely linked with disease stability. These results suggest that biomarkers of the NO pathway may provide valuable insights into COPD pathophysiology and could serve as supportive tools for disease monitoring. While ADMA and NO show promise as biomarkers for COPD exacerbations, further large-scale and longitudinal studies are warranted to assess their cost-effectiveness, standardization, availability, and potential role in guiding clinical management and therapeutic strategies across different clinical settings. Given the potential impact of inhaled therapies and PDE-4 inhibition on NO bioavailability and endothelial signaling, future prospective studies should capture detailed medication profiles to evaluate treatment–biomarker interactions. In addition, undiagnosed obstructive sleep apnea (OSA) may have influenced NO-related biomarkers in our study. OSA independently impairs endothelial function and reduces NO bioavailability; circulating NO derivatives are suppressed and may normalize with CPAP therapy, consistent with endothelial nitric oxide pathway dysfunction. Moreover, intermittent hypoxia and oxidative stress in OSA can modulate eNOS activity and the L-arginine/ADMA axis, potentially increasing ADMA and diminishing NO signaling [31]. By contrast, several studies report increased exhaled NO indices (e.g., FeNO or alveolar NO) in OSA, reflecting airway inflammation rather than improved systemic NO bioavailability [32]. Taken together, undetected OSA could bias serum ADMA/NO/eNOS levels toward higher ADMA and lower circulating NO, while exhaled NO may track differently. Future studies should incorporate OSA screening (e.g., questionnaires and/or sleep testing) to quantify and adjust for this confounder.

## Figures and Tables

**Table 1:** Demographic and clinical findings.

		^1^ Control (*n* = 50)	^2^ Stable COPD (*n* = 50)	^3^ Acute Exacerbation (*n* = 50)	*p*	^1−2^ *p*	^1−3^ *p*	^2−3^ *p*
**Age (Year)**	*Mean ± SD*	55.00 ± 6.93	53.30 ± 6.93	53.16 ± 7.30	*^a^* *0.351*	*^aa^* *0.690*	*^aa^* *0.583*	*^aa^* *1.000*
	*Median (Min–Max)*	56 (42–67)	52 (39–68)	54 (35–68)				
**Gender (F/M)**	**Female**	26 (52.0)	25 (50.0)	25 (50.0)	*^b^* *1.000*			
	**Male**	24 (48.0)	25 (50.0)	25 (50.0)				
**BMI**	*Mean ± SD*	23.99 ± 1.75	28.27 ± 3.53	29.37 ± 4.87	***^a^*** 0.001**	***^aa^*** 0.001**	***^aa^*** 0.001**	*^aa^* *0.403*
	*Median (Min–Max)*	24.7 (19.6–26.3)	28.1 (22.3–35.7)	28.5 (21.1–40.6)				
**Systolic blood pressure**	*Mean ± SD*	126.88 ± 19.08	125.56 ± 22.23	129.50 ± 6.53	*^a^* *0.357*	*^aa^* *1.000*	*^aa^* *1.000*	*^aa^* *0.770*
	*Median (Min–Max)*	126.3 (81.5–165.4)	126.3 (66.2–162.3)	129.5 (120–140)				
**Diastolic blood pressure**	*Mean ± SD*	77.62 ± 11.09	75.77 ± 9.35	94.40 ± 9.99	***^a^*** 0.001**	*^aa^* *1.000*	***^aa^*** 0.001**	***^aa^*** 0.001**
	*Median (Min–Max)*	78.8 (52.8–104.4)	74.9 (58.8–93)	96 (75–110)				
**FEV1 (% predicted)**	*Mean ± SD*	85.33 ± 2.13	52.61 ± 12.50	46.00 ± 13.22	***^a^*** 0.001**	***^aa^*** 0.001**	***^aa^*** 0.001**	***^aa^*** 0.031*
	*Median (Min–Max)*	85.2 (81.6–89.3)	50 (32.6–78.9)	47.9 (20.5–69.8)				
**FEV1/FVC**	*Mean ± SD*	83.30 ± 4.19	65.94 ± 6.46	56.58 ± 8.70	***^a^*** 0.001**	***^aa^*** 0.001**	***^aa^*** 0.001**	***^aa^*** 0.001**
	*Median (Min–Max)*	83 (76–90)	69 (42–70)	57.5 (38–70)				
**PaO_2_ (mmHg)**	*Mean ± SD*	87.26 ± 7.51	61.43 ± 7.73	57.43 ± 8.47	***^a^*** 0.001**	***^aa^*** 0.001**	***^aa^*** 0.001**	***^aa^*** 0.038*
	*Median (Min–Max)*	87.5 (75–100)	61.5 (43.7–73.5)	58 (42.9–71.2)				
**SaO_2_ (%)**	*Mean ± SD*	94.75 ± 1.64	80.05 ± 6.15	77.70 ± 6.35	***^a^*** 0.001**	***^aa^*** 0.001**	***^aa^*** 0.001**	*^aa^* *0.074*
	*Median (Min–Max)*	94.6 (91.9–97.6)	80 (68.2–91)	77.6 (65.4–91.3)				
pH	*Mean ± SD*	7.40 ± 0.03	7.35 ± 0.03	7.30 ± 0.03	***^a^*** 0.001**	***^aa^*** 0.001**	***^aa^*** 0.001**	***^aa^*** 0.001**
	*Median (Min–Max)*	7.4 (7.4–7.4)	7.4 (7.3–7.4)	7.3 (7.3–7.3)				
pCO_2_	*Mean ± SD*	40.06 ± 2.82	52.18 ± 4.44	63.79 ± 5.91	***^a^*** 0.001**	***^aa^*** 0.001**	***^aa^*** 0.001**	***^aa^*** 0.001**
	*Median (Min–Max)*	40 (35.5–44.8)	51.9 (45.5–59.7)	63.1 (55.2–73)				

^a^ One Way Anova Test, ^aa^ Bonferroni Test, ^b^ Pearson’s Chi Square Test; ^1^ Control group, ^2^ Stable COPD group, ^3^ Acute Exacerbation of COPD group; ** *p* < 0.01 * *p* < 0.05.

**Table 2:** Laboratory findings, serum ADMA, NO, and eNOS levels.

		^1^ Control (*n* = 50)	^2^ Stable COPD (*n* = 50)	^3^ Acute Exacerbation (*n* = 50)	*p*	^1−2^ *p*	^1−3^ *p*
**FBG**	*Mean ± SD*	86.74 ± 6.92	88.6 ± 11.39	95.52 ± 9.21	^a^0.001**	^aa^0.961	^aa^0.001**
	*Median (Min–Max)*	86.5 (75–99)	87.7 (69.1–122.5)	95 (80–110)			
**Insulin (μU/mL)**	*Mean ± SD*	9.96 ± 3.02	10.08 ± 3.26	9.72 ± 3.02	*^a^* *0.840*	*^aa^* *1.000*	*^aa^* *1.000*
	*Median (Min–Max)*	10 (5–15)	10 (5–15)	9.5 (5–15)			
**HOMA-IR**	*Mean ± SD*	2.13 ± 0.67	2.22 ± 0.86	2.29 ± 0.76	*^a^* *0.582*	*^aa^* *1.000*	*^aa^* *0.901*
	*Median (Min–Max)*	2.1 (1–3.4)	2.1 (1–4.5)	2.1 (1.1–4)			
**CRP (mg/L)**	*Mean ± SD*	1.20 ± 0.34	1.97 ± 1.37	1.05 ± 2.70	***^c^*** 0.001**	*^cc^* *0.330*	***^cc^*** 0.001**
	*Median (Min–Max)*	1.2 (0.6–1.8)	1.9 (0.1–5.2)	0.5 (0.1–19.3)			
**ESR (mm/h)**	*Mean ± SD*	7.34 ± 2.21	22.18 ± 9.69	40.58 ± 16.91	***^a^*** 0.001**	***^aa^*** 0.001**	***^aa^*** 0.001**
	*Median (Min–Max)*	7.2 (3–13)	21.8 (5–42.1)	40.2 (11.4–78.4)			
**Triglyceride (mg/dL)**	*Mean ± SD*	104.14 ± 15.62	105.4 ± 14.44	118.34 ± 21.42	***^a^*** 0.001**	*^aa^* *0.908*	***^aa^*** 0.001**
	*Median (Min–Max)*	104 (78–130)	105.5 (79–130)	115.5 (75–175)			
**LDL (mg/dL)**	*Mean ± SD*	99.69 ± 11.06	103.66 ± 12.85	139.41 ± 24.84	***^a^*** 0.001**	*^aa^* *0.228*	***^aa^*** 0.001**
	*Median (Min–Max)*	99.9 (75–118.8)	103.3 (75.6–133.2)	137.4 (96–184.6)			
**Total cholesterol (mg/dL)**	*Mean ± SD*	173.00 ± 10.82	172.70 ± 10.5	200.82 ± 24.61	***^a^*** 0.001**	***^aa^*** ***1.000***	***^aa^*** 0.001**
	*Median (Min–Max)*	172.5 (155–198)	172.5 (155–197)	199.5 (162–250)			
**HDL (mg/dL)**	*Mean ± SD*	52.48 ± 7.25	47.96 ± 5.42	37.74 ± 4.77	***^a^*** 0.001**	***^aa^*** 0.002**	***^aa^*** 0.001**
	*Median (Min–Max)*	52.5 (40–65)	48 (39–59)	38 (30–47)			
**Albumin (g/dL)**	*Mean ± SD*	42.78 ± 4.79	43.26 ± 4.08	39.06 ± 4.67	***^a^*** 0.001**	*^aa^* *1.000*	***^aa^*** 0.001**
	*Median (Min–Max)*	42.5 (35–50)	43.5 (35–50)	40.4 (28.1–52)			
**Total protein (g/dL)**	*Mean ± SD*	71.36 ± 6.06	70.24 ± 5.1	69.12 ± 7.07	*^a^* *0.191*	*^aa^* *1.000*	*^aa^* *0.207*
	*Median (Min–Max)*	70.5 (61–80)	70.5 (60–80)	68.5 (51.7–81)			
**ADMA (umol/L)**	*Mean ± SD*	1.17 ± 0.03	1.27 ± 0.03	1.61 ± 0.031	***^d^*** ***0*** **.** 001**	*^dd^* *0.085*	***^dd^*** 0.001**
	*Median (Min–Max)*	1.2 (1–1.4)	1.2 (0.9–2)	1.5 (1.3–2.1)			
**NO (μmol/L)**	*Mean ± SD*	18.52 ± 0.33	13.75 ± 0.3	11.07 ± 0.32	***^d^*** 0.001**	***^dd^*** 0.001**	***^dd^*** 0.001**
	*Median (Min–Max)*	18.4 (15–21.9)	13.6 (10.1–18)	11.1 (7.4–14)			
**eNOS (IU/mL)**	*Mean ± SD*	47.27 ± 0.62	43.97 ± 0.56	42.19 ± 0.58	***^d^*** 0.001**	***^dd^*** 0.001**	***^dd^*** 0.001**
	*Median (Min–Max)*	47.6 (40.2–55)	44.2 (35.3–49.9)	42 (35.1–51.3)			

^a^ One-Way Anova Test; ^aa^ Bonferroni Test, ^c^ Kruskal–-Wallis Test; ^cc^ Dunn Bonferroni Test, ^d^ ANOVA Test, ^dd^ Bonferroni Test (adjusted for BMI). ^1^ Control group, ^2^ Stable COPD group, ^3^ Acute Exacerbation of COPD group; ** *p* < 0.01.

**Table 3:** Correlation analysis between NO-related biomarkers and laboratory and clinical findings in COPD patients.

	ADMA (umol/L)		NO (μmol/L)		eNOS (IU/mL)	
	r	* p*	r	* p*	r	* p*
**Age (Year)**	−0.005	*0.959*	0.152	*0.132*	0.077	*0.446*
**•BMI**	0.265	0.001 **	−0.430	<0.001 ***	−0.330	<0.001 ***
**Duration of COPD**	−0.011	*0.910*	−0.062	*0.543*	−0.084	*0.407*
**Systolic blood pressure**	0.030	*0.765*	−0.103	*0.310*	−0.067	*0.511*
**Diastolic blood pressure**	0.394	<0.001 ***	−0.313	0.002 **	−0.097	*0.340*
**FEV1 (% predicted)**	−0.481	<0.001 ***	0.286	0.004**	0.372	<0.001 ***
**FEV1/FVC**	−0.293	0.003 **	0.120	*0.236*	0.086	*0.397*
**PaO_2_ (mmHg)**	−0.209	0.038 *	0.067	*0.511*	0.098	*0.337*
**SaO_2_ (%)**	−0.272	0.006 **	0.345	<0.001 ***	0.067	*0.512*
**FBG**	0.133	*0.189*	−0.312	0.002 **	−0.107	*0.292*
**Insulin (μU/mL)**	0.026	*0.798*	−0.024	*0.813*	0.072	*0.477*
**HOMA-IR**	0.057	*0.579*	−0.130	*0.199*	0.030	*0.766*
**CRP(mg/L)**	−0.106	*0.294*	0.130	*0.198*	−0.002	*0.982*
**ESR (mm/h)**	0.344	<0.001 ***	−0.282	0.005 **	−0.080	*0.430*
**Triglyceride (mg/dL)**	0.255	0.011 *	−0.177	*0.080*	−0.192	*0.057*
**LDL (mg/dL)**	0.512	<0.001 ***	−0.442	<0.001 ***	−0.210	0.037 *
**Total cholesterol (mg/dL)**	0.484	<0.001 ***	−0.392	<0.001 ***	−0.194	*0.054*
**HDL (mg/dL)**	−0.452	<0.001 ***	0.450	<0.001 ***	0.249	0.013 *
**Albumin (g/dL)**	−0.274	0.006 **	0.205	0.042 *	0.087	*0.394*
**Total protein (g/dL)**	−0.071	*0.488*	0.032	*0.754*	0.015	*0.879*
**pH**	−0.419	<0.001 ***	0.366	<0.001 ***	0.070	*0.488*
**PCO_2_**	0.393	<0.001 ***	−0.418	<0.001 ***	−0.211	0.036 *

r: Partial correlation coefficient (adjusted for BMI). r: Pearson’s correlation coefficient; *** *p* < 0.001 ** *p* < 0.01 * *p* < 0.05.

**Table 4:** Comparisons by gender and exacerbation frequency.

	ADMA (μmol/L)		NO (μmol/L)		eNOS (IU/mL)	
	Mean ± SD	Median (Min–Max)	Mean ± SD	Median (Min–Max)	Mean ± SD	Median (Min–Max)
**Gender (F/M)**						
**Female**	1.38 ± 0.031	1.3 (0.9–2.1)	14.12 ± 0.38	13.7 (7.4–21.9)	44.13 ± 0.49	43.8 (35.1–55)
**Male**	1.32 ± 0.031	1.3 (0.9–2)	14.77 ± 0.39	14 (8.1–21.5)	44.84 ± 0.49	44 (37.4–54.9)
**^d^** ***p***	*0.166*		*0.235*		*0.316*	
**Frequency of exacerbations**						
**0**	1.57 ± 0.05	1.5 (1.3–2)	11.19 ± 0.36	11 (8.4–13.9)	41.82 ± 0.64	41.1 (37.4–51.3)
**1**	1.72 ± 0.07	1.9 (1.3–2)	10.81 ± 0.57	10.1 (8.1–13.7)	41.55 ± 1.05	41.3 (37.1–46.7)
**≥2**	1.62 ± 0.08	1.4 (1.3–2.1)	11.16 ± 0.61	12 (7.4–14)	43.05 ± 1.10	44.1 (35.1–48)
**^d^** ***p***	*0.206*		*0.852*		*0.567*	

^d^ ANCOVA Test & Bonferroni Test (adjusted for BMI).

**Table 5:** ROC curve analysis for patients with acute exacerbation of COPD.

Variables	AUC (95% CI)	Cut-Off	*p* Value	Sensitivity (%95 CI)	Specificity (%95 CI)	PPV (%95 CI)	NPV (%95 CI)
**ADMA (umol/L)**	0.983 (0.934–0.998)	***≥1.36***	***<0.001***	86.0 (73.3–94.2)	96.0 (86.3–99.5)	95.6 (84.6–98.8)	87.3 (77.5–93.2)
**eNOS (IU/mL)**	0.823 (0.734–0.892)	***≤44.52***	***<0.001***	82.0 (68.6–91.4)	70.0 (55.4–82.1)	73.2 (63.7–81.0)	79.5 (67.7–87.8)

*DeLong Test*.

**Table 6:** ROC curve analysis for patients with stable COPD.

Variables	AUC (95% CI)	Cut-Off	*p* Value	Sensitivity (%95 CI)	Specificity (%95 CI)	PPV (%95 CI)	NPV (%95 CI)
**ADMA (umol/L)**	0.609 (0.506–0.705)	***≥1.36***	*0.062*	34.0 (21.2–48.8)	96.0 (86.3–99.5)	89.5 (67.4–97.2)	59.3 (54.2–64.1)
**NO (μmol/L)**	0.921 (0.850–0.966)	***≤14.91***	***<0.001***	68.0 (53.3–80.5)	100.0 (92.9–100.0)	100 (100.0–100.0)	75.8 (67.6–82.4)
**eNOS (IU/mL)**	0.713 (0.614–0.799)	***≤48.8***	***<0.001***	94.0 (83.5–98.7)	42.0 (28.2–56.8)	61.8 (55.9–67.5)	87.5 (69.0–95.6)

*DeLong Test*.

## Data Availability

The datasets used and/or analyzed during the current study are available from the corresponding author on reasonable request.
